# Comprehensive analysis of EMT-related genes and lncRNAs in the prognosis, immunity, and drug treatment of colorectal cancer

**DOI:** 10.1186/s12967-021-03065-0

**Published:** 2021-09-15

**Authors:** Yang Yang, Mingyang Feng, LiangLiang Bai, Weiting Liao, Kexun Zhou, Mengxi Zhang, Qiuji Wu, Feng Wen, Wanting Lei, Pengfei Zhang, Nan Zhang, Jiaxing Huang, Qiu Li

**Affiliations:** 1 Department of Medical Oncology, Cancer Center, West China Hospital, Sichuan University, No. 37, GuoXue Xiang, Chengdu, Sichuan China; 2 West China Biomedical Big Data Center, Sichuan University, No. 37, GuoXue Xiang, Chengdu, Sichuan China

**Keywords:** EMT, Colorectal cancer, Metastasis, Immunity, Treatment, Prognosis

## Abstract

**Background:**

EMT is an important biological process in the mechanism of tumor invasion and metastasis. However, there are still many unknowns about the specific mechanism of EMT in tumor. At present, a comprehensive analysis of EMT-related genes in colorectal cancer (CRC) is still lacking.

**Methods:**

All the data were downloaded from public databases including TCGA database (488 tumor samples and 52 normal samples) as the training set and the GEO database (GSE40967 including 566 tumor samples and 19 normal samples, GSE12945 including 62 tumor samples, GSE17536 including 177 tumor samples, GSE17537 including 55 tumor samples) as the validation sets. One hundred and sixty-six EMT-related genes (EMT-RDGs) were selected from the Molecular Signatures Database. Bioinformatics methods were used to analyze the correlation between EMT-RDGs and CRC prognosis, metastasis, drug efficacy, and immunity.

**Results:**

We finally obtained nine prognostic-related EMT-RDGs (FGF8, NOG, PHLDB2, SIX2, SNAI1, TBX5, TIAM1, TWIST1, TCF15) through differential expression analysis, Unicox and Lasso regression analysis, and then constructed a risk prognosis model. There were significant differences in clinical characteristics, 22 immune cells, and immune functions between the high-risk and low-risk groups and the different states of the nine prognostic-related EMT-RDGs. The methylation level and mutation status of nine prognostic-related EMT-RDGs all affect their regulation of EMT. The Cox proportional hazards regression model was also constructed by the methylation sites of nine prognostic-related EMT-RDGs. In addition, the expression of FGF8, PHLDB2, SIX2, and SNAIL was higher and the expression level of NOG and TWIST1 was lower in the non-metastasis CRC group. Nine prognostic-related EMT-RDGs also affected the drug treatment response of CRC.

**Conclusions:**

Targeting these nine prognostic-related EMT-RDGs can regulate CRC metastasis and immune, which is beneficial for the prognosis of CRC patients, improve drug sensitivity in CRC patients.

**Supplementary Information:**

The online version contains supplementary material available at 10.1186/s12967-021-03065-0.

## Background

Colorectal cancer (CRC) is a malignant tumor with high morbidity and mortality, easy recurrence, and easy metastasis. CRC is mostly asymptomatic and difficult to detect in the early stage, and most patients are already in the advanced stage when they are diagnosed [1]. Besides, advanced CRC infiltrates lymph nodes and is prone to metastasis of abdominal implantation or metastasis to other organs. The prognosis of the patient is very poorer and the survival rate is extremely lower [2]. Reducing the probability of CRC metastasis and finding new targets is the key to improving the survival of CRC patients.

Epithelial-mesenchymal transition (EMT) is one of the main mechanisms of tumor metastasis and invasion [3]. It also has the effect of promoting the malignant proliferation of tumor cells, reducing apoptosis and senescence, and promoting immune suppression. Loss of E-cadherin expression and loss of cell polarity are the key steps of EMT [4]. The main E-cadherin inhibitors that have been discovered are Snail, Zeb, E47, and KLF8, which combine with the promoter of E-cadherin and inhibit its expression. Twist, Goosecoid, E2.2, and FoxC2 indirectly inhibit the activity of E-cadherin. The three protein complexes (Par, Crumbs, and Scribble) that maintain apical-basal polarity in epithelial cells are also regulated by EMT-induced genes, and cell polarity is lost after inhibition [5]. TGF-β family, Wnts, Notch, EGF, HGF, FGF, HIF and other signaling pathways play an important role in regulating the above process. Therefore, the EMT process is also critical in the development, metastasis, and invasion of colorectal cancer.

EMT can affect the occurrence and development of CRC, the prognosis of metastasis, and the effect of chemotherapy and immunotherapy [6]. However, the current research still lacks systematic research on the overall genes that regulate the EMT process and its prognosis and treatment effects with CRC. Therefore, we use TCGA and GEO data as training and validation sets to screen out differentially expressed EMT-related genes (EMT-RDGs) and lncRNAs. Construct a prognostic model to study their relationship with the prognosis, immune infiltration, drug sensitivity, and resistance of CRC patients, and provide a basis for clinical treatment of CRC.

## Materials and methods

### Data collection and collation

All the data are downloaded from the TCGA database (https://portal.gdc.cancer.gov/) including 588 tumor samples and 48 normal samples as the training set and the GEO database (GSE40967 including 566 tumor samples and 19 normal samples, GSE12945 including 62 tumor samples, GSE17536 including 177 tumor samples, GSE17537 including 55 tumor samples) as the validation set. The data about liver metastasis of CRC was downloaded from GSE28814 (GPL13425) set including 125 tumor samples. The therapeutic data of CRC was obtained from the GSE36864 set including 349 tumor samples. Data types of TCGA include transcriptome, DNA methylation, mutation data, copy number variants (CNV), and clinical data. We searched “Epithelial-mesenchymal transition”, “Mesenchymal-epithelial transition” in the Molecular Signatures Database (http://www.gsea-msigdb.org/gsea/msigdb/search.jsp). We deleted the duplicated genes and left only one in all the gene sets. Finally, 166 EMT-related genes were selected from the Molecular Signatures Database. The EMT-RDGs were screened out from the training set and used WGCNA co-expression analysis to obtain EMT-related lncRNAs (EMT-RlncRNAs) and perform differential expression analysis. The correlation between EMT-RDGs is analyzed. The protein–protein interaction (PPI) network analysis is carried out on the String website (https://string-db.org/). The expression levels of EMT-RDGs between CRC with or without liver metastasis were analyzed in the GSE6988 dataset. The relationship between the expression levels of EMT-RDGs and therapy (capecitabine group, capecitabine + irinotecan group, and XELOX (capecitabine + oxaliplatin) + bevacizumab group) of advanced CRC was analyzed in the GSE36864 set.

### Gene enrichment and function analysis

The Webgestalt website (http://www.webgestalt.org/) is used for GO analysis (biological processes, cellular components, and molecular functions), KEGG signaling pathways based on EMT-RDGs.

### Gene mutation and methylation analysis

All genetic mutation landscapes are shown through waterfall diagrams. The prognostic mutations of EMT-RDGs were analyzed, and the gene mutation level was compared between wild-type and mutant. The mutation data includes analysis and summary of somatic variation using maftools [7]. Analysis of CNV was performed in the GSCA database (http://bioinfo.life.hust.edu.cn/web/GSCALite/). For each CRC patient, the tumor mutation burden (TMB) score is measured as follows: (total mutations/total covered bases) × 10^6^.

The methylation level of key prognostic EMT-RDGs was analyzed in the cBioPortal database (http://www.cbioportal.org), DNMIVD website [8] (http://119.3.41.228/dnmivd/index/) and MEXPRESS website (https://mexpress.be/).

### Cluster analysis of EMT-RDGs

K-means method [9] is used for unsupervised cluster analysis to classify CRC samples. Choose the K value corresponding to the largest delta area as the number of clusters to analyze the gene expression, clinical characteristics, and immune characteristics of each cluster. The cluster analysis was used R package “ConsensusClusterPlus”.

### Prognostic model construction based on EMT-RDGs and EMT-RlncRNAs

One thousand two hundred and eighty-nine tumor samples were included for the analysis of clinical pathology and prognosis. Univariate Cox regression analysis was used to screen out the prognostic-related EMT-RDGs. The Lasso regression model uses dimensionality reduction to calculate the score of each gene for constructing a prognostic model based on the EMT-RDGs related to the prognosis. The risk score calculation formula is: gene expression1*genecoef 1 + gene expression2*genecoef 2 + gene expression3*genecoef 3 +…+ gene expression N*genecoef N. Then univariate cox regression and multivariate cox regression analysis were used to analyze independent prognostic factors from CRC from clinical factors and gene expression, and the results were visualized by forest plots. The Nomogram model based on the multivariate Cox model is used to predict the risk and prognosis of CRC by obtaining the approximate probability value of the dependent variable according to the value of the predictor variable.

### Immune cell infiltration and immune microenvironment score

ESTIMATE (Estimation of STromal and Immune cells in Malignant Tumour tissues using Expression data) is used to calculate the purity of stromal cells and immune cells in the tumor microenvironment. CIBERSORT [9] is used to calculate the infiltration level of the main 22 immune cells. Their differences are compared in unsupervised clustering and prognostic risk models. In the TIMER database (http://timer.comp-genomics.org/), the immune cell enrichment level is calculated by the xCELL method, the immune cell infiltration level is also calculated by the EPIC method and the MCP-counter package, and the immune cells are quantitatively analyzed by QUANTISEQ. At the same time, the immune association between immune infiltrates and gene expression, the association between immune infiltrates and mutation status, somatic CNV, and clinical outcome are obtained in TIMER. The score of immune cells and functions was calculated by ssGSEAScore (“GSVA package” and “GSEABase package”) based on the transcriptome data of TCGA and GSE40967 data.

### Single-cell analysis

Single cells (Endothelial, Epithelial, Fibroblast) from 11 CRC patients were profiled using Fluidigm based single-cell RNA-seq protocol to characterized cellular heterogeneity of CRC (GSE81861).

### Drug therapy information

Prognostic-related drugs related to the expression of EMT-RDGs were screened in the GSCA online analysis platform (http://bioinfo.life.hust.edu.cn/web/GSCALite/). The effect of gene expression and mutation on drug resistance and sensitivity is analyzed on the CARE website (http://care.dfci.harvard.edu/).

### Verification of the expression level of EMT-RDGs

The expression levels of prognostic EMT-RDGs have been verified in the Oncomine database, the Human Protein Atlas (HPA) database (https://www.proteinatlas.org/), DNMIVD website [10] (http://119.3.41.228/dnmivd/index/) and GEPIA database (http://gepia2.cancer-pku.cn/#index).

### Statistical analysis

All data analysis and visualization are performed in R.4.2. All results are considered statistically significant with P < 0.05. The figures were shown by *P < 0.05, **P < 0.01, ***P < 0.001, and ****P < 0.0001.

## Results

### Differential expression analysis of EMT-related genes and lncRNA

We downloaded a total of 588 CRC samples and 48 normal samples from the TCGA database. Combined with clinical data, after removing incomplete data, we finally got 429 CRC samples for subsequent analysis. We have obtained 56 EMT-RDGs including 40 genes with high expression and 16 genes with low expression and analyzed the association between them. Most of the genes are related to each other (Additional file 1: Figure S1).

### Gene enrichment and function analysis

**Fig. 1:**
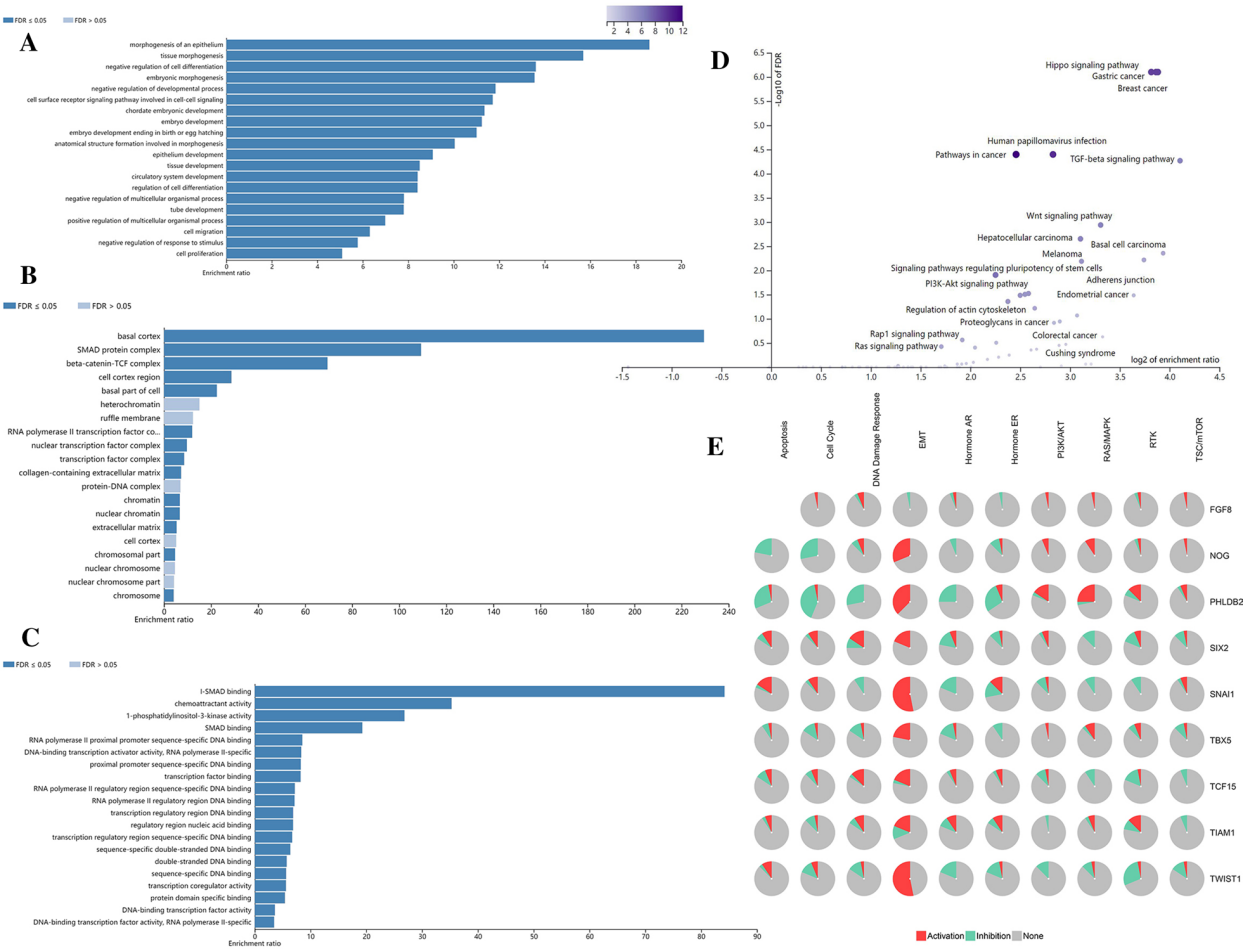
The gene function enrichment of EMT-RDGs in CRC from Webgestalt. **A** EMT-RDGs enrichment ratio in biological process. **B** EMT-RDGs enrichment ratio in cellular components. **C** EMT-RDGs enrichment ratio in cellular components. **D** The KEGG signaling pathway analysis of EMT-RDGs. **E** The function analysis of prognosis-related genes

GO analysis showed that EMT-RDGs focus on epithelial morphogenesis, tissue morphogenesis, negative regulation of cell proliferation, and other processes in the biological process; (Fig. 1A) the cellular components focused on the base cortex, SMAD protein complex, beta-catenin-TCF-complex, and so on; (Fig. 1B) the molecular functions focus on I-SMAD binding, chemoattractant activity, 1-phosphatidylinositol-3-kinase activity, and other functions (Fig. 1C). The KEGG signaling pathway is indeed mainly enriched in TGF-β, Hippo, Wnt signaling pathways, and other mechanisms (Fig. 1D). The special function analysis of prognosis-related EMT-RDGs verified that these key EMT-RDGs were indeed EMT-related genes (Fig. 1E).

### Cluster analysis of EMT-RDGs

We divided the CRC samples into two clusters according to the principle of unsupervised cluster classification. The samples with BMI ≥ 23.5 [BMI = weight (Kg)/height^2^(m^2^)] were more in cluster2 and the remaining samples with BMI < 23.5 are more in cluster1. Other clinical characteristics, overall survival, and 22 immune cells were not significant differences in the two clusters (Additional file 1: Figure S2).

### Prognostic model construction based on EMT-RDGs

**Fig. 2:**
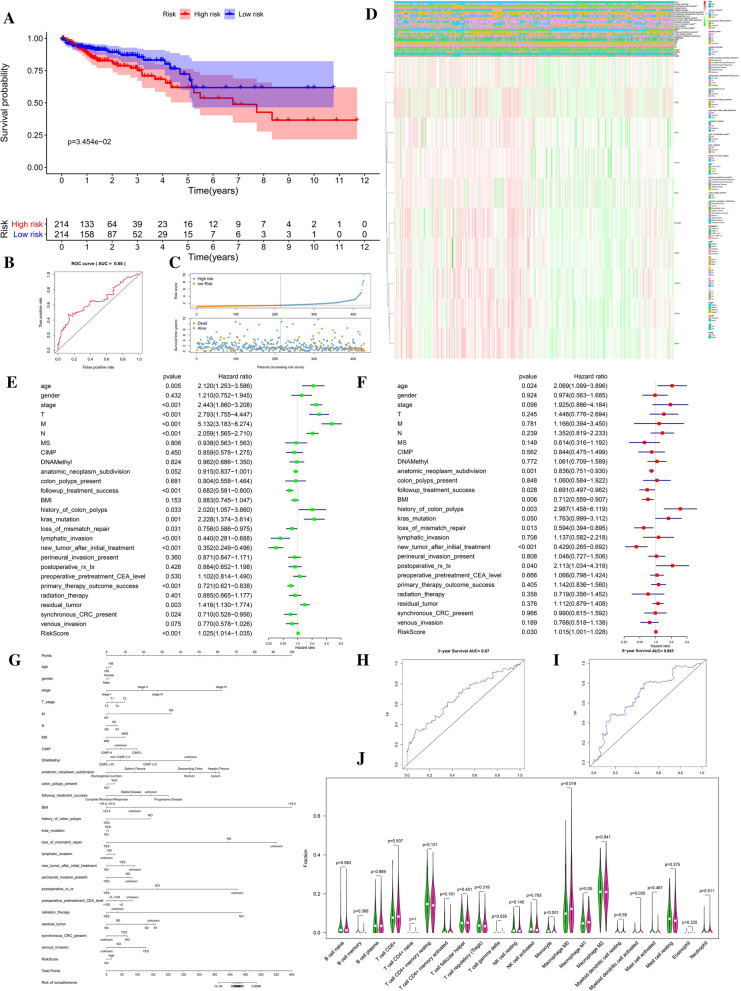
Risk prognosis model construction of nine prognostic EMT-RGDs in TCGA data by unicox and lasso regression. **A** Survival curve comparing high-risk and low-risk groups by R package “survival”. **B** ROC curve of risk sore by R package “survivalROC”. **C** The distribution of risk score and the scatterplot of the relationship between risk scores and survival time by R package “ggplot”. **D** Heat map of prognostic EMT-RDGs and clinical parameters at high risk and low risk groups by R package “pheatmap”. **E** The univariate cox forest map of the clinical characteristics in the training set by R package “survival” and “forestplot”. **F** The multivariate cox forest plot of the clinical characteristics in the training set by R package “survival” and “forestplot”. **G** The nomogram baseline of multivariate cox analysis by R package “rms”. **H** ROC curve of 3-year survival. **I** ROC curve of 5-year survival by R package “survivalROC”. **J** 22 types of immune cells infiltration of high risk and low risk group in TCGA data by R package “e1071”, “parallel” and “preprocessCore”. *P < 0.05, **P < 0.01, ***P < 0.001, and ****P < 0.0001

**Fig. 3:**
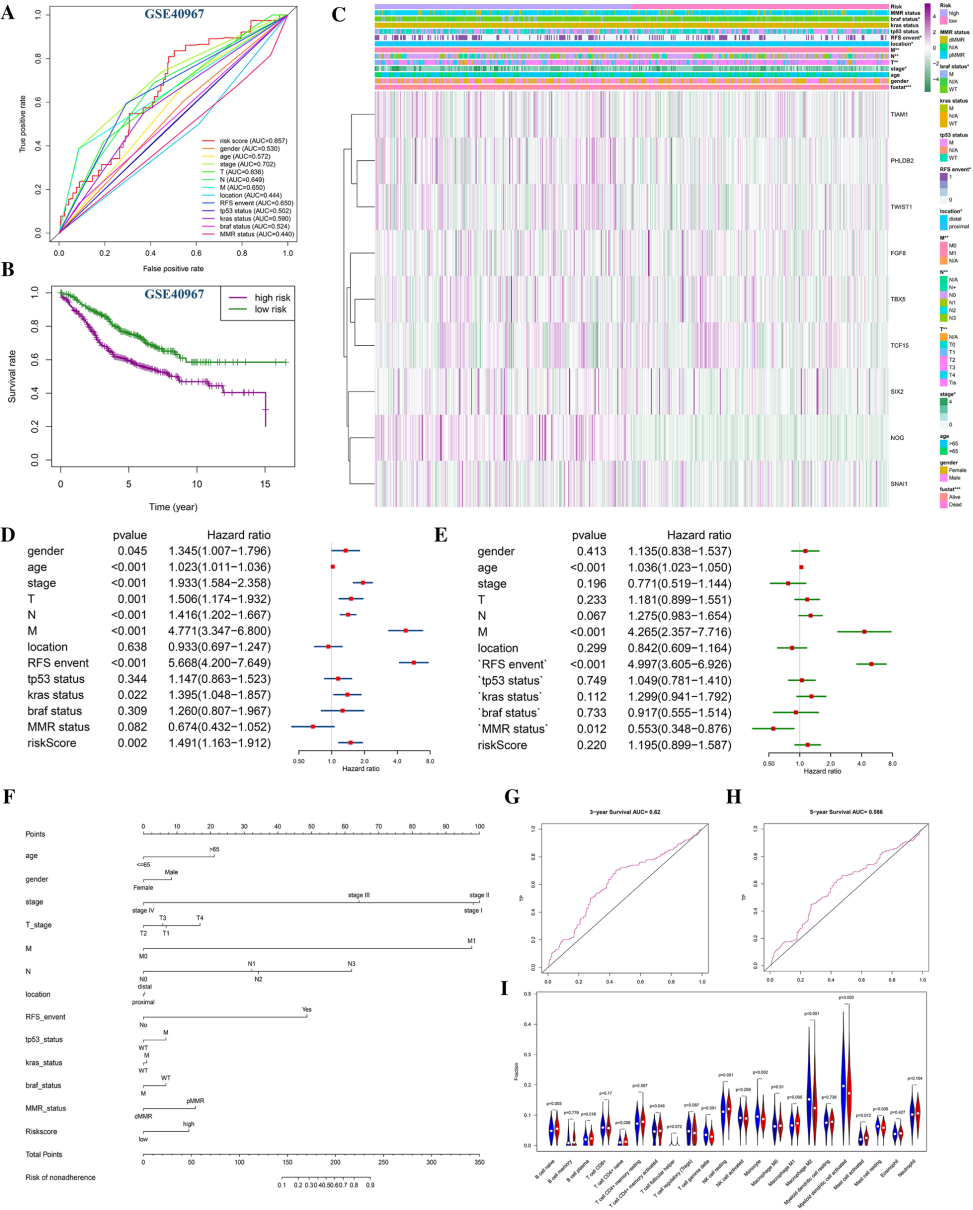
Risk prognosis model verification of nine prognostic EMT-RGDs in GSE40967 data. **A** ROC curve of risk sore and other clinical characteristics in GSE40967 data by R package “survivalROC”. **B** Survival curve comparing high-risk and low-risk groups by R package “survival”. **C** Heat map of prognostic EMT-RDGs and clinical parameters at high risk and low-risk groups by R package “pheatmap”. **D** The univariate cox forest map of the clinical characteristics in the training set by R package “survival” and “forestplot”. **E** The multivariate cox forest plot of the clinical characteristics in the training set by R package “survival” and “forestplot”. **F** The nomogram baseline of multivariate cox analysis by R package “rms”. **G** ROC curve of 3-year survival (P < 0.05). **H** ROC curve of 5-year survival by R package “survivalROC” (P < 0.05). **I** 22 types of immune cells infiltration of high risk and low-risk group in TCGA data by R package “e1071”, “parallel” and “preprocessCore”. *P < 0.05, **P < 0.01, ***P < 0.001, and ****P < 0.0001

Nine prognostic-related EMT-RDGs were screened by univariate cox and then Lasso regression analysis in the TCGA data, and the score of each gene was calculated. The risk prognosis model of CRC was constructed by the expression level of each gene*risk score. The effect of the model was verified by substituting the corresponding value of GEO data into the following formula: (Riskscore = TCF15*0.006387445 + SIX2*0.000957825 + NOG*0.016976643 + FGF8*0.047052635 + TBX5*0.00178245 + SNAI1*0.000456714 + PHLDB2*1.08E-05 + TIAM1*6.55E-05 + TWIST1*6.70E-05). The model was verified by substituting the corresponding value of GEO data into the above formula. The overall survival (OS) was longer in low-risk group of TCGA training set (Fig. 2A and C), GSE40967 (HR = 0.54857, 95% CI 0.41328–0.72814) (Fig. 3B), GSE12954 set (HR = 0.576808, 95% CI 0.184833–1.800043) (Additional file 1: Figure S3A, B), GSE17536 set (HR = 0.587008, 95% CI 0.370944–0.928924) (Additional file 1: Figure S3E, F) and GSE17537 set (HR = 0.032210, 95% CI 0.013055–0.079467) (Additional file 1: Figure S3I, J). The disease-free survival (DFS) was also longer in the low-risk group of GSE12954 set (p > 0.05) **(**Additional file 1: Figure S3C), GSE17536 set (p < 0.05) (Additional file 1: Figure S3G) and GSE17537 set (p < 0.05) (Additional file 1: Figure S3K). The area under the ROC curve (AUC) was 0.66 in TCGA (Fig. 2B), 0.657 in GSE40967 (Fig. 3C), 0.639 in GSE17536 (Additional file 1: Figure S3H), and 0.854 in GSE17537 (Additional file 1: Figure S3L), respectively, indicating that this model had good accuracy in predicting the prognosis of CRC patients. However, the validation of the GSE12954 set (Additional file 1: Figure S3D) showed a meaningless result. The model predicted the 3-year survival rate more accurately (Figs. 2H, 3G), but the accuracy of the 5-year survival rate was average (Figs. 2I, 3H). In the TCGA data, pathological staging, TNM staging, follow-up treatment success, BMI, history of colon polyps, dMMR, permanent invasion present, primary therapy outcome success, synchronous colon cancer present, and venous invasion are all significantly different in high and low-risk groups (Fig. 2D). More than 65 years of age, history of colon polyps, KRAS gene mutation, new tumors after initial treatment were found to be risk factors for the prognosis of CRC, and dMMR is a protected factor by the univariate and multivariate cox regression. Advanced clinical and pathological stage, residual tumor, and high-risk score are only risk factors for the prognosis of CRC, and non-invasive lymph nodes and successful primary treatment are protective factors in univariate regression (Fig. 2E). Distal bowel segment, BMI ≥ 23.5, and postoperative_rx_tx found only in multivariate regression were risk factors (Fig. 2F).

In the GEO data, > 65-year-old, M stage, and RFS events are also risk factors that are regressed by univariate and multivariate cox. Male, pathological stage, clinical TN stage, KRAS gene mutation, and risk score were found to be risk factors in univariate regression (Fig. 3D). In multivariate cox regression, MMR is a protective factor (Fig. 3E).

In addition, the Nomogram model constructed based on multivariate cox regression is a tool used to predict the prognostic risk of CRC (Figs. 2G, 3F).

### Construction of a prognostic model based on EMT-RlncRNAs

32 EMT-RlncRNAs were analyzed by WGCNA in the TCGA dataset (Additional file 1: Figure S4A). We also constructed another prognostic risk model with EMT-RlncRNAs (Riskscore = AC068418.2*0.000119527496121585 + AC006273.1*0.0076287254340738 + LINC02437*0.00163200945364475 + LINP1*0.0120094796161845 + GPC5.IT1*0.000613250610676045). The AUC of ROC was 0.585 (Additional file 1: Figure S4B). The survival trend was longer in the low-risk group (Additional file 1: Figure S4C). The perineural and lymphatic invasion was strongly correlated to the prognostic risk of CRC (Additional file 1: Figure S4D). More than 65-year-old, advanced TNM stage, history of colon polyps, KRAS mutation, lymphatic invasion, new tumor after initial treatment, and residual tumor were risk factors; and pMMR and synchronous CRC present were protective factors in univariate regression (Additional file 1: Figure S4E). In multivariate cox regression, over 65-year-old, history of colon polyps, and KRAS mutation were also risk factors; anatomic neoplasm subdivision, BMI < 23.5, pMMR, and new tumor after initial treatment were protective factors (Additional file 1: Figure S4F). The nomogram model was constructed by multivariate cox regression (Additional file 1: Figure S4G).

### Nine prognosis-related EMT-RDGs and prognosis of CRC

In TCGA, only the highly expressed FGF8 corresponds to a longer survival time (Additional file 1: Figure S5A). In the GSE40967 set, the low expression of NOG, SIX2, and SNAI1 correspond to a longer survival time (Additional file 1: Figure S5B). In the GEPIA database, low expression of NOG, PHLDB2, SIX2, SNAI1, and TCF15 has a better prognosis in CRC (Additional file 1: Figure S5C). The verification in the GCSC database found that the high expression of TBX5, TCF15, NOG, SIX2, and SNAI1 in CRC patients has a higher survival risk (Additional file 1: Figure S5D).

### The relationship between prognostic-related EMT-RDGs and immunity

**Fig. 4:**
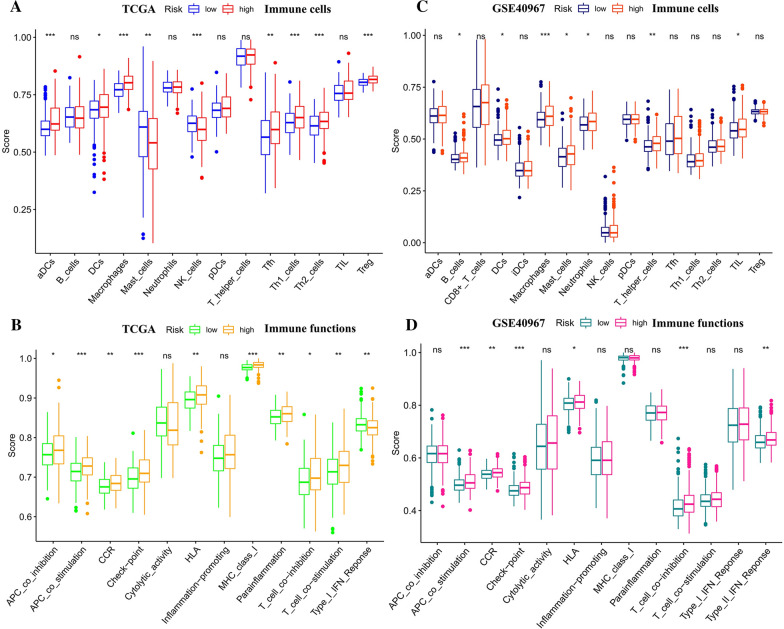
The comparable score of immune cells and functions based on the prognostic risk model. **A** The score of immune cells comparing high-risk and low-risk groups by ssGSEAScore in TCGA data. **B** The score of immune functions comparing high-risk and low-risk groups by ssGSEAScore in TCGA data. **C** The score of immune cells comparing high-risk and low-risk groups by ssGSEAScore in GSE40967 data. **D** The score of immune functions comparing high-risk and low-risk groups by ssGSEAScore in GSE40967 data. *P < 0.05, **P < 0.01, ***P < 0.001, and ****P < 0.0001

Based on the CIBERSORT algorithm in TCGA data, monocytes had higher infiltration levels in the high-risk group, and Macrophage M0, Macrophage M1, and activated DCs had higher infiltration levels in the low-risk group (Fig. 3J). In GSE40967 data, T cell gamma delta, Monocyte, Macrophage M2, activated dendritic cell and mast cell resting were higher in the high-risk group; while B cell naive, B cell plasma, T cell CD4 + naive, and T cell CD4 + Memory resting, T cell CD4 + memory activated, NK cell resting, Macrophage M1 and Mast cell activated have higher levels in the low-risk group (Fig. 3I). The score of DCs, macrophages, Tfh cells, Th1 cells, Th2 cells, and Tregs was significantly higher in the high-risk group, and the score of mast cells and NK cells was higher in the low-risk group from the TCGA set (Fig. 4A). In the GSE40967 set, the score of B cells, DCs, macrophages, neutrophils, Th cells, and TIL was higher in the high-risk group. However, the score of mast cells was higher in the high-risk group (Fig. 4B). The score of immune functions including co-stimulation or co-suppression of antigen-presenting cells (APC), chemotaxis of CCR, immune checkpoints, HLA, parainflammation, MHC class I, and T cell activation co-stimulation or co-suppression was higher in the high-risk group. The score of Type I INF response was higher in the low-risk group in the TCGA set (Fig. 4C). In the GSE40967 set, the score of APC co-stimulation, CCR, checkpoints, HKA, T cell co-suppression, and Type II INF response was higher in the high-risk group (Fig. 4D).

In TIMER, we found that nine genes had different effects on immune cell infiltration, and thus had different effects on immunotherapy. Highly expressed FGF8 was positively correlated with the infiltration level of CD4 + T cells, CD8 + T cells, activated NK cells, macrophages M1, cancer-associated fibroblasts, and B cells; and was negatively correlated with the infiltration level of hematopoietic stem cells, neutrophil, resting NK cells and macrophages M0. The infiltration level of immune cells (p < 0.05) was usually more abundant in mutated-type FGF8 CRC than wild-type FGF8. The statistically significant results of immune cells were lower in arm-level deletion CNA of CRC compared to normal CNA (Additional file 1: Figure S6-aA–C). The effect of gene expression combined with the level of immune cell infiltration on the prognosis of CRC had been focused on. The survival time was longer in low FGF8 expression + low cancer associated fibroblast, high FGF8 expression + low common lymphoid progeniters, high FGF8 expression + low eosinophils, high FGF8 expression + high macrophage M0, high FGF8/NOG expression + low macrophage M2, low FGF8 expression + low cancer associated fibroblast, low FGF8 expression + high Tfh cells, low FGF8 expression + high γδT cells and high FGF8 expression + high T memory resting cells (Additional file 1: Figure S6-aD); in high NOG expression + low eosinophils, high NOG expression + low cancer associated fibroblast, low NOG expression + low B cell memory, and high NOG expression + high CD8 + T cells (Additional file 1: Figure S6-b); in high PHLDB2 expression + low B cell naive, low PHLDB2 expression + high endothelial cells, low PHLDB2 expression + low resting mast cells, high PHLDB2 expression + high neutrophils and high PHLDB2 expression + high CD4 + T cells (Additional file 1: Figure S6-c); in high SIX2 expression + high B cell naive, low SIX2 expression + high activated NK cells, low SIX2 expression + low T CD4 + naive cells, high SIX2 expression + high CD4 + T cells and low SIX2 expression + low Tfh cells (Additional file 1: Figure S6-d); in high SNAI1 expression + low B cells, high SIX2 expression + low eosinophil cells, low SNAI1 expression + high macrophage M1 cells, high SNAI1 expression + low Tfh cells and low SIX2 expression + high CD8 + T cells (Additional file 1: Figure S6-e); in high TBX5 expression + high B plasma cells, low TBX5 expression + high macrophage M0 cells, high TBX5 expression + low macrophage M2 cells, high TBX5 expression + low activated mast cells, high TBX5 expression + low resting DC cells, low TBX5 expression + low NK cells and high TBX5 expression + high T cell CD4 + memory activated (Additional file 1: Figure S6-f); in high TCF15 expression + high B plasma cells, low TCF15 expression + low activated DC cells, high TCF15 expression + low resting DC cells and high TCF15 expression + low neutrophils (Additional file 1: Figure S6-g); in high TIAM1 expression + low B cells, low TIAM1 expression + high macrophage M1 cells, low TIAM1 expression + low resting mast cells and low TIAM1 expression + high T cell CD4 + memory activated (Additional file 1: Figure S6-h); in high TWIST1 expression + low B cells, low TWIST1 expression + low B cell memory and low TWIST1 expression + low eosinophil (Additional file 1: Figure S6-i).

### Single-cell analysis verification

After discovering that nine EMT-RDGs have a significant correlation with stromal cells and immune cells in the CRC microenvironment, we further explored the heterogeneity and function of these cells in CRC to verify whether they are related to EMT. In the single-cell sequencing data set (GSE81861), PHLDB2 was positively correlated to cancer-associated fibroblasts (CAFs) about the EMT state of CRC (Additional file 1: Figure S7).

### The correlation between nine prognosis-related EMT-RDGs and methylation

**Fig. 5:**
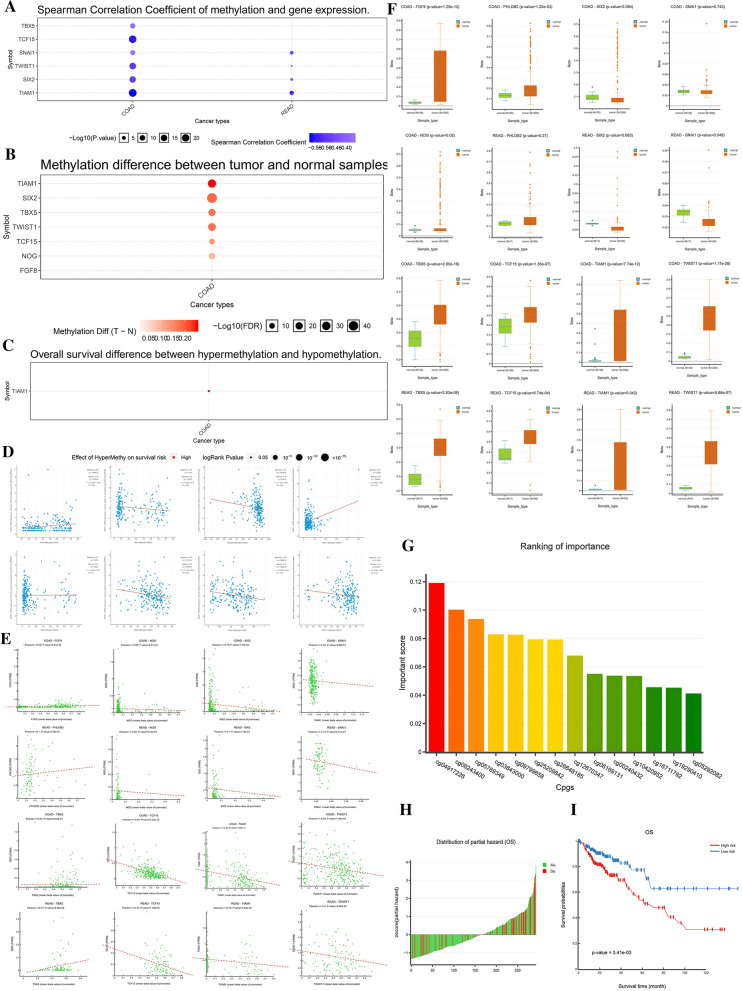
The methylation analysis of nine prognostic EMT-RGDs in CRC. **A** The correlation between methylation and gene expression of the prognostic EMT-RGDs with spearman analysis from GCSC database. **B** The differential analysis of methylation level about the prognostic EMT-RGDs between tumor and normal tissues from GCSC database. **C** Overall survival difference between hypermethylation and hypomethylation of TIAM1 from GCSC database. **D** The effect of hypermethylation on survival risk about the prognostic EMT-RGDs from cBioPortal database. **E** The correlation between methylation and gene expression of the prognostic EMT-RGDs with Pearson analysis from the DNMIVD database. **F** The differential analysis of methylation level about the prognostic EMT-RGDs between tumor and normal tissues from DNMIVD database. **G** The important CpG methylation sites of nine prognostic EMT-RGDs from DNMIVD database. **H** The Cox proportional hazards regression model was based on the important CpG methylation sites of nine prognostic EMT-RGDs from the DNMIVD database. **I** Survival curve comparing high-risk and low-risk groups by R package “survival”

Next, we tested the methylation status and mutation level of nine prognostic-related EMT-RDGs on the prognosis of CRC, as well as their correlation with drug response and resistance. The expression of TBX5, TIAM1, SIX2, TWIST1, SNAI1, and TCF15 was negatively correlated to methylation **(**Fig. 5A). The expression of FGF8 and SNAI1 was significantly positively correlated with methylation level in the GCSC database; while the expression of NOG, PHLDB2, TBX5, TIAM1, and TWIST1 was negatively correlated with methylation level from the cBioPortal database (Fig. 5D). In DNMIVD analysis, the expression of FGF8, PHLDB2, and TBX5 was verified that was significantly positively correlated with methylation. The remaining prognosis-related EMT-RDGs were negatively correlated with methylation (Fig. 5E).

In the GCSC database, the methylation levels of FGF8, NOG, TCF15, TWIST1, TBX5, SIX2, and TIAM1 are higher in colon cancer (Fig. 5B). The hypermethylation of TIAM1 has a higher survival risk for colon cancer but lacking data in rectal cancer. The remaining genes did not have effective information (Fig. 5C). In the DNMIVD database, the methylation level of FGF8, NOG, PHLDB2, TCF15, TWIST1, TBX5, and TIAM1 was higher in CRC compared to normal tissues. On the contrary, the methylation level of SIX2 and SNAI1 was higher in CRC (Fig. 5F).

The top five CpG methylation sites associated with nine prognosis-related EMT-RDGs that were identified were cg04917226, cg06243400, cg05769349, cg03843000, and cg09799658 (Fig. 5G). All the CpG methylation sites were shown in Additional file 1: Table S1. The Cox proportional hazards regression model (Fig. 5H) was constructed based on CpG methylation sites associated with Nine prognosis-related EMT-RDGs and indicated longer OS in the low-risk group (Fig. 5I).

### The relationship between the expression and mutation of nine prognosis-related EMT-RDGs

**Fig. 6:**
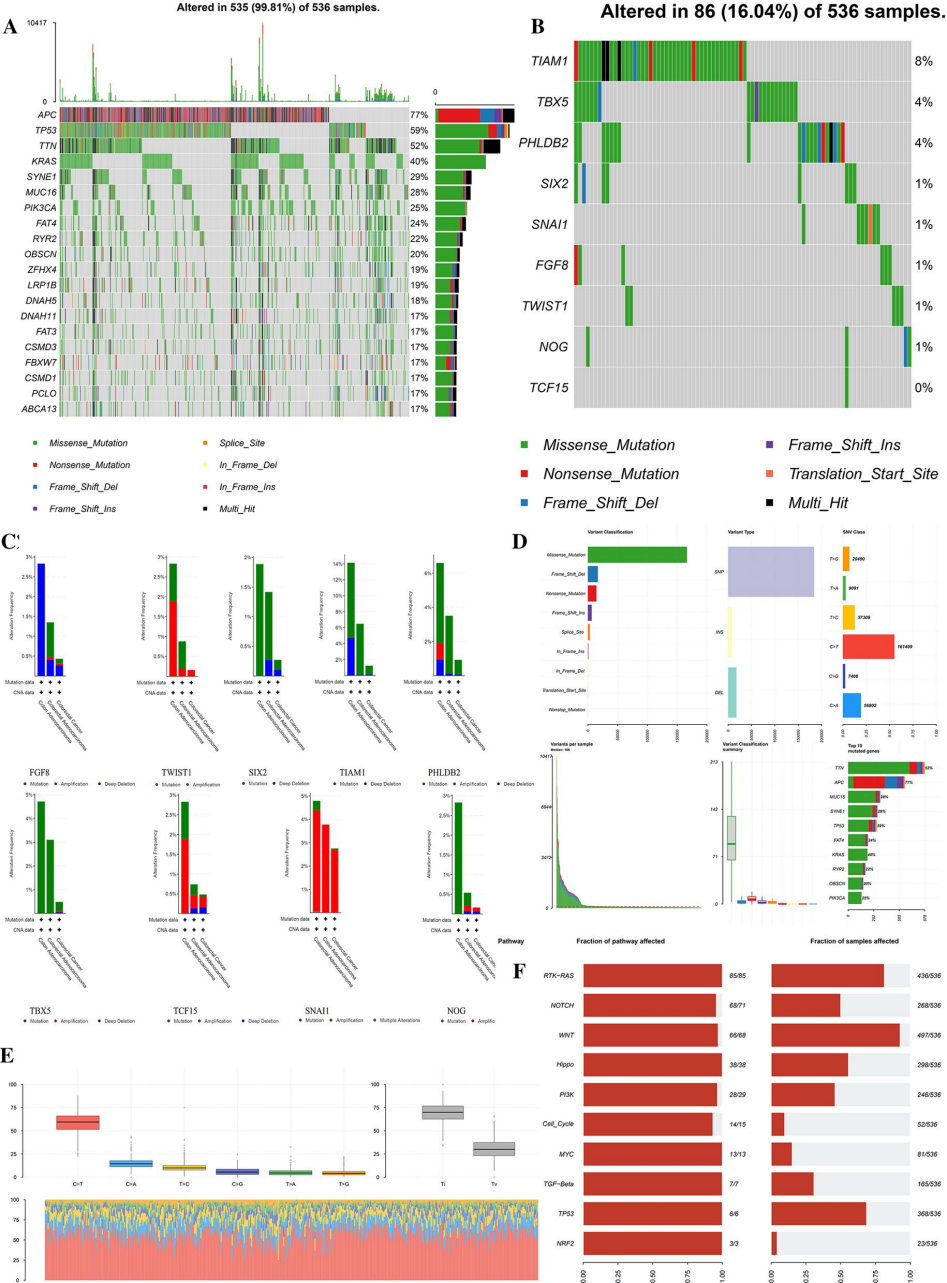
The mutation analysis of nine prognostic EMT-RGDs in CRC. **A** The oncoplot of top genes with mutation rates in CRC by R package “maftools”. **B** The oncoplot of nine prognostic EMT-RGDs with mutation rates in CRC by R package “maftools”. **C** The distribution of mutation types about nine prognostic EMT-RGDs in three types of CRC from cBioPortal database. **D** The variation classification and type of the prognostic EMT-RGDs in CRC from cBioPortal database. **E** The comparison of SNV class from cBioPortal database. **F** The fraction of pathway affected by the gene mutation by R package “maftools”

We analyzed the mutational panorama of the CRC gene and nine prognostic-related EMT-RDGs. The top five genes with mutation rates in CRC were APC, TP53, TTN, KRAS, and SYNE1 (Fig. 6A). The mutation rate of nine prognosis-related EMT-RDGs is less than 10% in all samples from TCGA data (Fig. 6B). Nine prognostic EMT-RGDs were more likely to be mutated in adenocarcinoma of CRC from cBioPortal database. The frequency of copy number variation was relatively higher in FGF8, TWIST1, SNAI1, and TCF15 (Fig. 6C). The most frequent occurrence in single nucleotide variation (SNV) is C > T (Fig. 6D, E). The RTK-RAS and Wnt pathway were easily affected by gene mutation of CRC (Fig. 6F). In the GCSC database, The SNV frequency of TIAM1 altered in 60 samples was 50%; The SNV frequency of PHLDB2 was 27%; The SNV frequency of TBX5 was 23%; The SNV frequency of FGF8 was 10% (Additional file 1: Figure S8A). The mutation frequency of TIAM1 in colon cancer was 29%. The mutation frequency of PHLDB2 in CC was 18%. The mutation frequency of TBX5 in CC was 17%. The mutation frequency of other prognostic-related EMT-RDGs in CC and all prognostic-related EMT-RDGs in rectal cancer was less than 10% (Additional file 1: Figure S8B). In the cBioPortal database, the correlation between mutated count and fraction genome altered of nine prognostic EMT-RDGs and the comparison of expression Z-score of nine prognostic-related EMT-RDGs in the mutated and wild group were respectively shown in Additional file 1: Figure S9A, B. Among them, mutated types of were FGF8, NOG, SIX2, SNAI1, TIAM1, and TBX5 were enriched with missense. The mutated sites of these genes were shown in Additional file 1: Figure S9C. The comprehensive comparison of mutated counts and disease-free survival (DFS) of nine prognostic-related EMT-RDGs was analyzed in Additional file 1: Figure S9D.

**Fig. 7:**
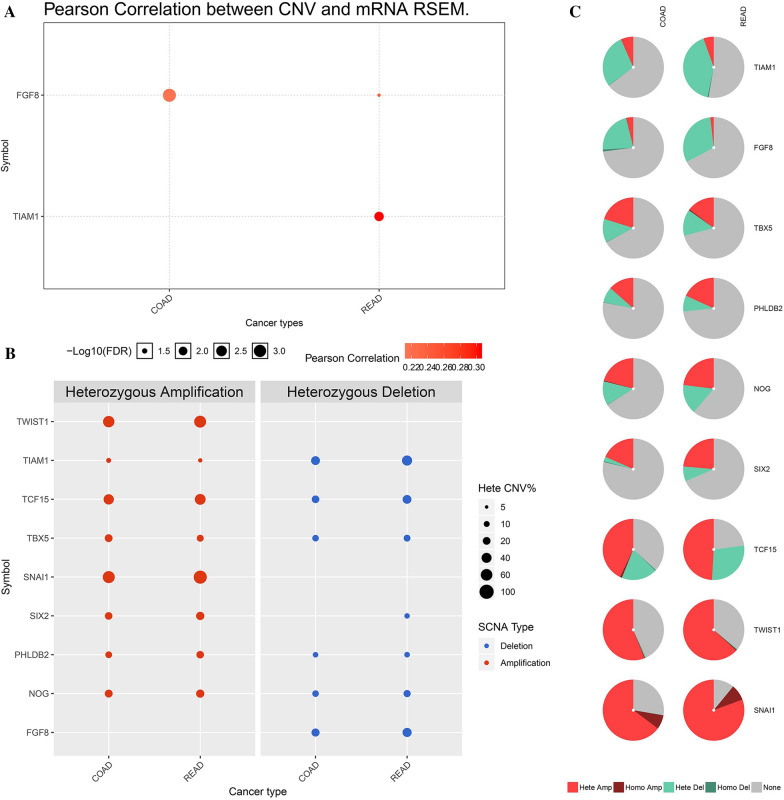
The CNV analysis of nine prognostic EMT-RGDs in CRC. **A** The correlation between CNV and gene expression of the prognostic EMT-RGDs with spearman analysis from GCSC database. **B** The heterozygous amplification and deletion about the nine prognostic EMT-RGDs in colon adenocarcinoma and rectal adenocarcinoma from GCSC database. **C** The distribution of CNV types about nine prognostic EMT-RGDs in CRC from GCSC database

The CNV of FGF8 in CRC was positively correlated to mRNA RSEM of FGF8. CNV of TIAM1 only in rectal cancer was significantly correlated to mRNA RSEM of TIAM1 (Fig. 7A). Heterozygous amplification of eight genes is present in CRC except for FGF8. Except for the heterozygous deletion of TWIST1 and SNAI1 in CRC, and the lack of SIX2 in colon cancer, all other genes have heterozygous deletions (Fig. 7B, C). The CNV status of nine prognostic-related EMT-RDGs was shown in Additional file 1: Figure S10.

### The relationship of prognosis-related EMT-RDGs and CRC metastasis

**Fig. 8:**
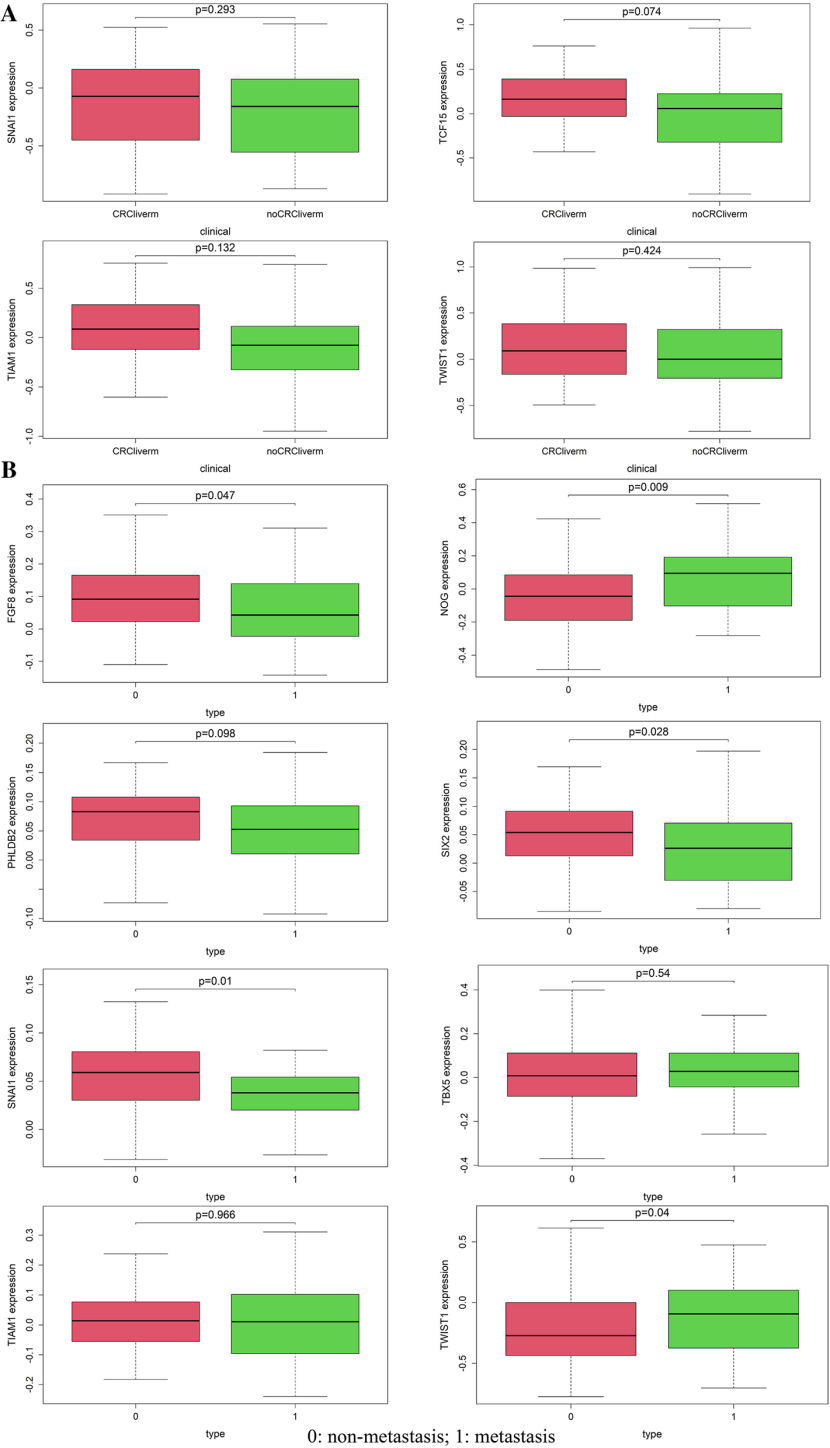
The expression analysis of nine prognostic EMT-RGDs in CRC with or without metastasis. **A** The expression level of nine prognostic EMT-RGDs between liver metastasis and non-metastasis of CRC in the GSE6988 dataset. **B** The expression level of nine prognostic EMT-RGDs between metastasis and non-metastasis of CRC in the GSE28814 (GPL13425) set (0: non-metastasis, 1: metastasis)

Twenty nine CRC with liver metastasis samples, 53 CRC without liver metastasis samples, 28 normal CRC samples, and 13 normal liver samples were obtained from the GSE6988 dataset. SNAI1, TCF15, TIAM1, and TWIST1 were found in this dataset. TCF15, TIAM1, and TWIST1 were significantly different in the four types of tissues (Fig. 8A). Although the expression of SNAI1, TCF15, TIAM1, and TWIST1 did not have a significantly statistical difference in the CRC with or without liver metastasis, the level of four EMT-RDGs was a higher trend in CRC with liver metastasis.

In GSE28814 (GPL13425) set, we got 92 non-metastasis CRC samples and 33 metastasis CRC samples. The expression of FGF8, PHLDB2, SIX2, and SNAIL was higher and the expression of NOG and TWIST1 was lower in the non-metastasis CRC group (Fig. 8B).

### The relationship of nine prognosis-related EMT-RDGs and CRC therapy

In the GSE36864 set, the expression of SIX2 was highest in CRC patients treated with capecitabine, followed in the capecitabine + irinotecan group, and finally in XELOX (capecitabine + oxaliplatin) + bevacizumab group. The remaining prognostic-related EMT-RDGs did not differ significantly among the three treatment groups. Moreover, the trend of FGF8, NOG, PHLDB2, and TIAM1 was consistent with the expression of SIX2 in three treatment groups (Additional file 1: Figures S10-S11).

**Fig. 9:**
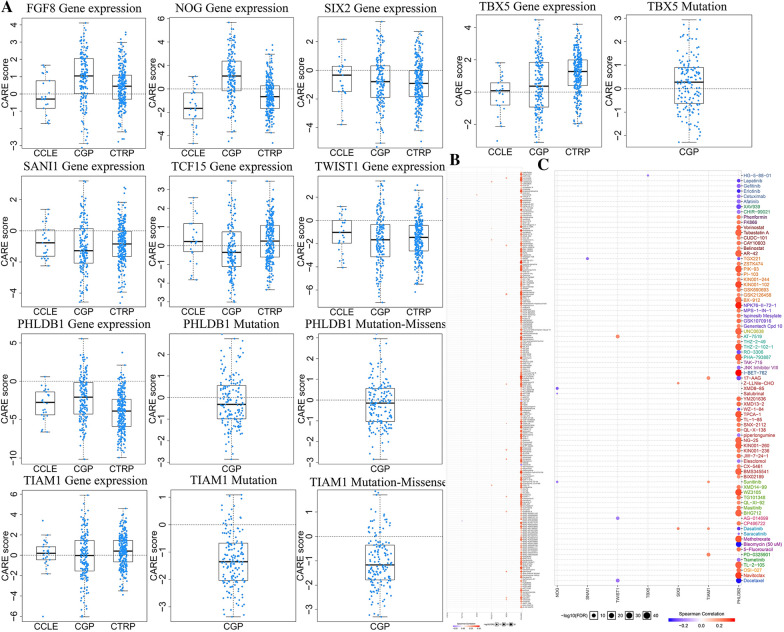
The drug sensitivity analysis of nine prognostic EMT-RGDs in CRC. **A** Computational analysis of resistance with nine prognosis-related EMT-RDGs of CRC in the CARE database. **B** The drug screen results of nine prognostic EMT-RGDs of CRC in the CTRP database. **C** The drug screen results of nine prognostic EMT-RGDs of CRC in the GDSC database

Computational analysis of resistance with nine prognosis-related EMT-RDGs in CRC showed a correlation with drug resistance and reactivity in the CARE database. The expressions and mutations of nine prognosis-related EMT-RDGs are mainly related to the reactivity and resistance of PI3K signaling pathway inhibitors and RAS/RAF/MEK/MAP signaling pathway inhibitors. Among them, PHLDB2 mutation is related to ZSTK474 resistance. The TBX5 mutation is related to the sensitivity of two BRAF_V600E mutation inhibitors: PLX4720 and 878739-06-1 (Fig. 9A).

In the CTRP database, FGF8 was related to tozarsetib resistance; TCF15 was related to BRD-K75293299 resistance; SIX2 were related to response sensitivity of trametinib; TWIST1 was positively correlated with treatment response of COL-3, skepinone-L, SR8278, and valdecoxib treatment sensitivity are related; vorinostat, SCH79797, Panobinostat, KX2-391, GSK-J4, entinostat, dinaciclib, CHM-1, brefeldin-A, belinostat, apicidin, and alvocidib. PHLDB2 is related to the non-response of JW-55 and dasatinib and the sensitivity of multiple drugs (Fig. 9B). In the GDSC database, NOG was negatively correlated with therapeutic sensitivity of Sunitinib, Salubrinal, and XMD8-85; SNAI1 was negatively correlated with TGX221 sensitivity; TWIST1 is negatively correlated with Docetaxel, AG-014699, and was positively correlated with AT-7519. TBX5 was negatively correlated with the sensitivity of HG-5-88-01; SIX2 was positively correlated with the sensitivity of Z-LLNle-CHO and Dasatinib; TIAM1 was positively correlated with the sensitivity of PD-0325901, Dasatinib, Sunitinib, and 17-AAG. PHLDB2 was positively correlated with the sensitivity of 5-Fluorouracil and negatively with the sensitivity of Gefitinib, Afatinib, Cetuximab, piperlongumine, Bleomycin (50 uM), and Docetaxel (Fig. 9C). All the information about drugs was shown in Additional file 1: Table S2.

### Verification of the expression level of EMT-RDGs in oncomine, GEPIA, and HPA database

**Fig. 10:**
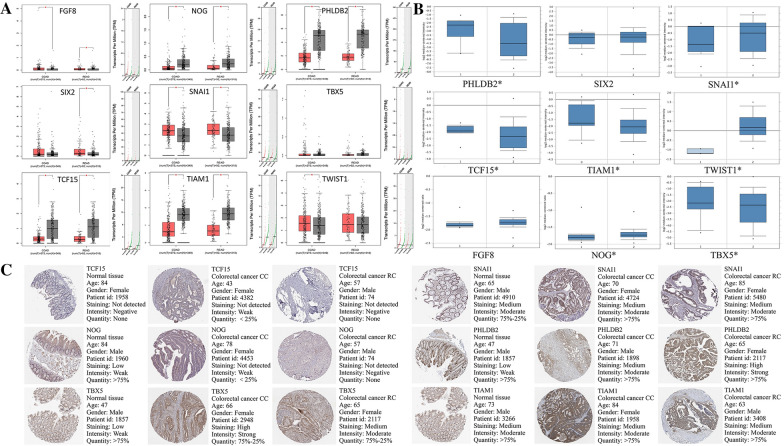
The expression verification of nine prognostic EMT-RGDs in CRC. **A** The expression verification of nine prognostic EMT-RGDs in the GEPIA database. **B** The expression verification of nine prognostic EMT-RGDs in the Oncomine database. **C** The immunohistochemistry of nine prognostic EMT-RGDs in the HPA database

In the TCGA database, the expression of TIAM1, PHLDB2, NOG, and TCF15 is low; the expression of SNAI1, FGF8, TWIST1, SIX2, and TBX5 is high in CRC. We verified the expression levels of nine prognostic-related EMT-RDGs in the GEPIA database. The high expression of FGF8, SIX2, SNAI1, and TWIST1 in CRC, and the low expression of NOG, PHLDB2, TCF15, and TIAM1 are consistent with our results. However, there is no significant difference in the expression of TBX5 (Fig. 10A). In the Oncomine database, the results of PHLDB2, SIX2, SNAI1, TCF15, TIAM1, TWIST1, and FGF8 are consistent with ours. However, the expression of NOG and TBX5 was contrary to our findings (Fig. 10B). In the HPA database, the results of the six genes exist but the information of FGF8, SIX2, and TWIST1 did not exist (Fig. 10C). In the DONIVD database, the results were consistent with the training set (Additional file 1: Figure S12).

## Discussion

The prone to invasion and metastasis of CRC is one of the main factors leading to poor prognosis of patients. EMT is one of the core mechanisms of tumor invasion and metastasis, and it also promotes tumor cell proliferation [11]. Therefore, we constructed a prognostic risk model for nine prognostic-related EMT-RDGs screened in the TCGA and GEO datasets and evaluated the reliability of the model and its relationship with survival and immunity. At the same time, we also analyzed the relationship between the expression, mutation, methylation of nine key EMT-RDGs and survival, immunity, and drug treatment response.

The transcription factor TCF15 has been found to affect the proliferation and differentiation of many types of cells, such as promoting hematopoietic stem cell quiescence and long-term self-renewal, [12] inducing the proliferation and differentiation of embryonic stem cells [13]. However, we found that TCF15 is low expressed in CRC, which may reduce the number of epithelial cells and promote the occurrence and metastasis of EMT of CRC.

miR-185 targeted SIX2 inhibiting the growth of HCC cells and the progression of EMT provides a new target for molecular therapy of liver malignancies [14]. Therefore, the potential of SIX2 in other tumor EMT is obvious, but its role in the CRC is not yet clear. We found that SIX2 is highly expressed in CRC, and reducing the expression will prolong the survival time of CRC.

NOG is one of the key genes of mesenchymal-epithelial interaction [15]. Studies have found that NOG disorders are related to the survival risk of nasopharyngeal carcinoma [16]. Increased expression of NOG significantly promotes breast cancer bone metastasis [17]. We found that low-expressed NOG has a good prognosis in CRC, indicating that NOG has the potential to be used in the treatment of CRC and even other tumors.

FGF8 is a mesodermal marker gene. When it is highly expressed, EMT is up-regulated and cell polarity is lost [18]. In tumors, high expression of FGF8 affects EMT through the BRG1/Snai1/E-cadherin pathway and promotes tumor proliferation and invasion of gastric cancer [19]. However, the high expression of FGF8 predicts a good prognosis in our results, which is contrary to the results for gastric cancer, and further research is needed to clarify its role.

The role of TBX5 in the EMT process is mainly to initiate the formation of mesenchymal limb progenitors [20]. Up-regulation of TBX5 promotes the formation of mesoderm during EMT and affects the differentiation of cardiomyocytes [21]. The increase of TBX5 drives the mesenchymal phenotype of breast cancer, promotes the EMT process, and inhibits the expression of the immune response network [22]. The high expression of TBX5 in our results indicates a high risk of poor prognosis.

SNAIL1 is a well-known tumor EMT inducer, and its role in CRC is relatively clear. The high expression of SNAIL has been found to induce a poor prognosis of CRC due to the induction of EMT phenotype. And SNAIL affects the EMT process of CRC through various mechanisms such as β-Catenin-LEF1 complexes [23]. Down-regulation of SNAIL1 mediated MYB and ISC markers (such as WiNTRLINC1) may help reduce EMT-related proliferation of CRC cells [24]. These studies are consistent with our findings.

PHLDB2 has been proven to be a downstream effector of the EMT pathway, and it may be an important biomarker and target for a good prognosis of CRC when its expression is low [25]. This is completely consistent with our results.

The overexpression of TIAM1 in lung adenocarcinoma is significantly related to advanced tumor grade and poor prognosis [26]. Knockout of TIAM1 expression can reverse the proliferation, migration, and EMT transformation of HCC cells [27]. The high expression of TIAM1 induced CRC proliferation and migration [28]. We found that the low expression of TIAM1 in CRC showed a good prognosis in the TCGA data but a poor prognosis in the GEO data, but the difference was not significant and there was no statistical significance. Therefore, further verification is required.

TWIST is also a clear inducer of EMT and mesenchymal phenotypic marker. Reducing its expression in CRC promotes the increase of E-calonectin and reverses the EMT process [29]. This is the same as our findings. It further illustrates the potential of TWIST as a CRC target and prognostic marker.

We found that nine key EMT-RDGs were closely associated with the metastasis of CRC, and first proposed that TBX5, FGF8, NOG, SIX2, and TCF15 are the role and potential of EMT-RDGs as prognostic markers and therapeutic targets in CRC. But it lacks in vivo experimental verification. Moreover, there were still few studies on the role of these genes' methylation and mutations in tumors. Therefore, we also studied the strong relationship and effects of the methylation and mutation status of these genes on the expression and prognosis. Targeting and monitoring the mutation status and methylation sites of these genes is also a potential tool to improve the prognosis of CRC. The EMT-RlncRNAs were selected to construct a prognostic model based on the TCGA data but fail to verify in GEO data due to the limited sample size.

EMT also promotes tumor immunosuppression. The current research on the relationship between immune cell infiltration and EMT mainly focuses on cancer-related fibroblasts, tumor-related macrophages, and EMT. TAM secretes a variety of cytokines and chemokines and promotes the paracrine transformation of adjacent epithelial tumor cells to EMT. In turn, the cytokines produced by tumor cells also promote the differentiation process of TAM, thereby forming a positive feedback loop between TAM and EMT in the process of tumor metastasis to promote tumor progression, invasion, and metastasis [30]. TAM induces the EMT program by regulating the JAK2/STAT3/miR-506-3p/FoxQ1 axis to enhance CRC migration, invasion, and CTC-mediated metastasis [31]. M2 macrophages promote the invasion and metastasis of lung cancer through EMT by up-regulating the expression of CRYAB and activating the ERK1/2/Fra-1/slug signaling pathway [32]. Inhibition of M2 macrophages inhibits EMT and fibrosis of CRC [33]. The high infiltration of M2 macrophages in the TCGA and GEO database was correlated to a high risk of prognosis and the high infiltration of M1 macrophages was correlated to a low risk of prognosis in CRC. However, research on other immune cells and EMT is still very few. When EMT decreases in CRC, CD14 + monocytes and CD19 + B cells also decrease, and the tumor increases infiltration of CD56 + NK cells [34].

Highly infiltrating CD8 + tumor-infiltrating lymphocytes in CRC are accompanied by a decrease in SNAIl and an increase in E-cadherin expression, which are closely related to EMT, and are closely related to the good prognosis of CRC [35]. However, in our results, the level of CD + 8 cell infiltration did not differ significantly between the high and low-risk groups. This may be due to the limited number of samples. EMT activation makes CRC cells more susceptible to NK cell-mediated NKG2D-mediated killing [36]. We indeed found that the level of resting NK cells increased in the low-risk group. Therefore, increasing the proportion of NK cells could improve the prognosis of CRC patients.

In addition, the relationship between the non-response or response of targeted drugs and nine prognostic-related EMT-RDGs expression or mutations was analyzed. These mainly include anti-angiogenesis targeted drugs, PI3K-Akt-mTOR signaling pathway, and RAS/RAF/MAPK signaling pathway targeted drugs. We found that these prognostic-related EMT-RDGs are closely related to drug treatment response. 5-FU is the basic chemotherapeutic drug for CRC, and cetuximab is the standard targeted drug for the first-line treatment of RAS/RAF wild-type left semi-CRC. PHLDB2 is positively correlated with 5-FU sensitivity, and it is negatively correlated with cetuximab sensitivity. These findings may help enhance drug sensitivity, reverse CRC resistance to prolong PFS and OS.

Some of these drugs have been confirmed in clinical trials that the survival benefit of CRC patients. For example, Sorafenib did not show superior therapeutic effects in CRC (RESPECT trial) [37]. Dabrafenib had shown a confirmed response rate in metastatic CRC with BRAF^V600E^-mutation positive (NCT01750918) [38]. Therefore, PLX4720, SB590885, 878739-06-1, and GDC-0879 are all potential drugs for BRAF^V600E^-mutant CRC. Most PI3K-Akt signaling pathway drugs for the targeted therapy of CRC are still in the research stage of in vivo, in vitro and I phase of clinical trials. However, some of these drugs have not been studied or performed in a clinical trial about CRC, so they can be considered as a targeted therapy option for colorectal cancer patients in the future. Meanwhile, the nine hub EMT-RDGs might be the potential biomarkers of targeted therapy response, also could predict the effectiveness of targeted drugs and synergy of genes and drugs.

## Conclusion

In summary, the nine vital EMT-RDGs are not only powerful prognostic markers and therapeutic targets for CRC, but also can be used as a key link in the occurrence of EMT caused by immune cells infiltrated in the tumor microenvironment, affecting the growth, invasion, and metastasis of CRC. They are expected to become a new target for targeted therapy and immunotherapy in the future.

## Supplementary Information

**Additional file 1: Figure S1.** The correlation of EMT-RDGs in CRC. **Figure S2. **The cluster analysis based on EMT-RDGs of CRC in TCGA set. (**A**) Consensus matrix of cluster analysis in CRC. (**B**) The CDF curve of cluster analysis in CRC. (**C**) Heat map of prognostic EMT-RDGs and clinical parameters at two clusters by R package “pheatmap”. (**D**) Survival curve comparing cluster 1 and 2 by R package “survival”. (**E**) 22 types of immune cells infiltration of two clusters in TCGA data by R package “1071”, “parallel” and “preprocessCore”. *P < 0.05, **P < 0.01, ***P < 0.001, and ****P < 0.0001. **Figure S3.** Risk prognosis model verification of 9 prognostic EMT-RGDs in three GEO data. (**A**) Overall survival curve comparing high-risk and low-risk groups by R package “survival” in the GSE12954 set. (**B**) The distribution of risk score and the scatterplot of the relationship between risk scores and overall survival time by R package “ggplot” in the GSE12954 set. (**C**) Disease-free survival curve comparing high-risk and low-risk groups by R package “survival” in the GSE12954 set. (**D**) ROC curve of risk sore and other clincial characteristics in the GSE12954 set by R package “survivalROC”. (**E**) Overall survival curve comparing high-risk and low-risk groups by R package “survival” in the GSE17536 set. (**F**) The distribution of risk score and the scatterplot of the relationship between risk scores and overall survival time by R package “ggplot” in the GSE17536 set. (**G**) Disease-free survival curve comparing high-risk and low-risk groups by R package “survival” in the GSE17536 set. (**H**) ROC curve of risk sore and other clincial characteristics in the GSE17536 set by R package “survivalROC”. (**I**) Overall survival curve comparing high-risk and low-risk groups by R package “survival” in the GSE17537 set. (**J**) The distribution of risk score and the scatterplot of the relationship between risk scores and overall survival time by R package “ggplot” in the GSE17537 set. (**K**) Disease-free survival curve comparing high-risk and low-risk groups by R package “survival” in the GSE17537 set. (**L**) ROC curve of risk sore and other clincial characteristics in the GSE17537 set by R package “survivalROC”. **Figure S4.** Risk prognosis model construction of the prognostic EMT-RlncRNAs in the TCGA data by unicox and lasso regression. (**A**) The network diagram of EMT-RlncRNAs by R packege “WGCNA”. (**B**) ROC curve of risk sore by R package “survivalROC”. (**C**) The overall survival curve comparing high-risk and low-risk groups by R package “survival”. (**D**) Heat map of prognostic EMT-RlncRNAs and clinical parameters at high risk and low risk groups by R package “pheatmap”. (**E**) The univariate cox forest map of the clinical characteristics in the training set by R package “survival” and “forestplot”. (**F**) The multivariate cox forest plot of the clinical characteristics in the training set by R package “survival” and “forestplot”. (**G**) The nomogram baseline of multivariate cox analysis by R package “rms”. *P < 0.05, **P < 0.01, ***P < 0.001, and ****P < 0.0001. **Figure S5.** The prognosis of 9 prognostic EMT-RGDs in CRC. (**A**) The overall survival curve comparing high-expression and low-expression of 9 prognostic EMT-RGDs in TCGA set by R package “survival”. (**B**) The overall survival curve comparing high-expression and low-expression of 9 prognostic EMT-RGDs in the GSE40967 set by R package “survival”. (**C**) The overall survival curve comparing high-expression and low-expression of 9 prognostic EMT-RGDs in the GEPIA database. (**D**) The effects of high expression of 9 prognostic EMT-RGDs on survival risk in the GCSC database. *P < 0.05, **P < 0.01, ***P < 0.001, and ****P < 0.0001.** Figure S6-a.** The relationship between the status of FGF8 and immune cells in CRC from TIMER database. (**A**) The correlation between the expression of FGF8 and the immune cell infiltration in CRC. (**B**) The comparism of immune cells infiltration in wild-type and mutated-type of FGF8 in CRC. (**C**) The comparism of immune cells infiltration in different CNA types of FGF8 in CRC. (**D**) The cumulative survival of the expression level of FGF8 and the immune cells infiltration in CRC. **Figure S6-b.** The relationship between the status of NOG and immune cells in CRC from TIMER database. (**A**) The correlation between the expression of NOG and the immune cell infiltration in CRC. (**B**) The comparism of immune cells infiltration in wild-type and mutated-type of NOG in CRC. (**C**) The cumulative survival of the expression level of NOG and the immune cells infiltration in CRC. **Figure S6-c.** The relationship between the status of PHLDB2 and immune cells in CRC from TIMER database. (**A**) The correlation between the expression of PHLDB2 and the immune cell infiltration in CRC. (**B**) The comparism of immune cells infiltration in wild-type and mutated-type of PHLDB2 in CRC. (**C**) The cumulative survival of the expression level of PHLDB2 and the immune cells infiltration in CRC. **Figure S6-d.** The relationship between the status of SIX2 and immune cells in CRC from TIMER database. (**A**) The correlation between the expression of SIX2 and the immune cell infiltration in CRC. (**B**) The comparism of immune cells infiltration in wild-type and mutated-type of SIX2 in CRC. (**C**) The cumulative survival of the expression level of SIX2 and the immune cells infiltration in CRC. **Figure S6-e.** The relationship between the status of SNAI1 and immune cells in CRC from TIMER database. (**A**) The correlation between the expression of SNAI1 and the immune cell infiltration in CRC. (**B**) The comparism of immune cells infiltration in wild-type and mutated-type of SNAI1 in CRC. (**C**) The comparism of immune cells infiltration in different CNA types of SNAI1 in CRC. (**D**) The cumulative survival of the expression level of SNAI1 and the immune cells infiltration in CRC. **Figure S6-f.** The relationship between the status of TBX5 and immune cells in CRC from TIMER database. (**A**) The correlation between the expression of TBX5 and the immune cell infiltration in CRC. (**B**) The comparism of immune cells infiltration in wild-type and mutated-type of TBX5 in CRC. (**C**) The cumulative survival of the expression level of TBX5 and the immune cells infiltration in CRC** Figure S6-g.** The relationship between the status of TCF15 and immune cells in CRC from TIMER database. (**A**) The correlation between the expression of TCF15 and the immune cell infiltration in CRC. (**B**) The comparism of immune cells infiltration in different CNA types of TCF15 in CRC. (**C**) The cumulative survival of the expression level of TCF15 and the immune cells infiltration in CRC. **Figure S6-h.** The relationship between the status of TIAM1 and immune cells in CRC from TIMER database. (**A**) The correlation between the expression of TIAM1 and the immune cell infiltration in CRC. (**B**) The comparism of immune cells infiltration in wild-type and mutated-type of TIAM1 in CRC. (**C**) The cumulative survival of the expression level of TIAM1 and the immune cells infiltration in CRC. **Figure S6-i.** The relationship between the status of TWIST1 and immune cells in CRC from TIMER database. (**A**) The correlation between the expression of TWIST1 and the immune cell infiltration in CRC. (**B**) The comparism of immune cells infiltration in wild-type and mutated-type of TWIST1 in CRC. (**C**) The cumulative survival of the expression level of TWIST1 and the immune cells infiltration in CRC. **Figure S7. **Single-cell analysis of 11 CRC patients in the GSE81861 set. (**A**) The function heat map of single-cell analysis. (**B**) The correlation between EMT and expression of PHLDB2. **Figure S8. **The mutation verification analysis of 9 prognostic EMT-RGDs in CRC from the GCSC database. (**A**) The SNV frequency of 9 prognostic EMT-RGDs based on the 60 CRC patients. (**B**) The mutation frequency of 9 prognostic EMT-RGDs in colon adenorcarcinoma and rectal adenorcarcinoma. **Figure S9. **The mutation verification analysis of 9 prognostic EMT-RGDs in CRC from the Cbiportal database. (**A**) The SNV frequency of 9 prognostic EMT-RGDs based on the 60 CRC patients. (**B**) The comparism of expression Z-score of the prognostic EMT-RGDs in mutated-type and wild-type. (**C**) The mutated sites of 9 prognostic EMT-RGDs. **D.** The comprehensive comparison of mutated counts and disease-free survival of 9 prognostic-related EMT-RDGs. **Figure S10. **The CNA verification analysis of 9 prognostic EMT-RGDs in CRC from the Cbiportal database. **Figure S11. **The expression comparism of 9 prognostic EMT-RGDs in CRC treated with capecitabine, capecitabine + irinotecan, and XELOX (capecitabine+oxaliplatin) + bevacizumab group in the GSE36864 set. **Figure S12. **The expression verification of 9 prognostic EMT-RGDs in the DONIVD database. **Table S1. **All the CpG sites and DNA methylation status of 9 prognostic EMT-RGDs from DNMIVD database. **Table S2. **All the drugs information of 9 prognostic EMT-RGDs from GDSC database.

## Data Availability

All data generated or analyzed during this study are included in this published article.

## Abbreviations

EMT: Epithelial-mesenchymal transition
CRC: Colorectal cancer
TCGA: The Cancer Genome Atlas
CNV: Copy number variation
OS: Overall survival
DFS: Disease-free survival
EMT-RDGs: Differentially expressed EMT-related genes
SNV: Single nucleotide variation
